# Recent Experiences and Advances in Contrast-Enhanced Subharmonic Ultrasound

**DOI:** 10.1155/2015/640397

**Published:** 2015-05-18

**Authors:** John R. Eisenbrey, Anush Sridharan, Ji-Bin Liu, Flemming Forsberg

**Affiliations:** ^1^Department of Radiology, Thomas Jefferson University, 132 South 10th Street, Philadelphia, PA 19107, USA; ^2^Department of Electrical and Computer Engineering, Drexel University, Philadelphia, PA 19104, USA

## Abstract

Nonlinear contrast-enhanced ultrasound imaging schemes strive to suppress tissue signals in order to better visualize nonlinear signals from blood-pooling ultrasound contrast agents. Because tissue does not generate a subharmonic response (i.e., signal at half the transmit frequency), subharmonic imaging has been proposed as a method for isolating ultrasound microbubble signals while suppressing surrounding tissue signals. In this paper, we summarize recent advances in the use of subharmonic imaging *in vivo*. These advances include the implementation of subharmonic imaging on linear and curvilinear arrays, intravascular probes, and three-dimensional probes for breast, renal, liver, plaque, and tumor imaging.

## 1. Introduction

Contrast-enhanced ultrasound (CEUS) relies on intravenously injected gas microbubbles to improve backscattering from within the vasculature [1]. Due to differences in acoustic impedance and compressibility between the microbubbles and surrounding media, ultrasound contrast agents act as nonlinear scatters. Relative to the transmit frequency (*f*_0_), this nonlinearity results in the generation of higher harmonics (*n* · *f*_0_), as well as ultraharmonics (*n*/2 *f*_0_). Contrast-specific software is now available on most ultrasound scanners to better visualize ultrasound contrast agents relative to the surrounding tissue [1]. The majority of these approaches rely on receiving signals at the higher harmonic frequency, thus suppressing linear signals from the surrounding tissue [1]. However, tissue has also been shown to generate nonlinear harmonic signals and thus complete tissue suppression using such techniques is rarely achieved [2].

Our group has focused on the development and application of subharmonic imaging (SHI) for over 15 years [3]. SHI works by receiving at half the transmitting frequency (*f*_0_/2) where tissue does not generate a nonlinear response. This technique benefits from increased depth penetration (due to less attenuation of the signal at the lower frequency) and improved contrast-to-tissue ratios (CTRs) relative to harmonic imaging [2]. The feasibility of SHI has been demonstrated for a variety of applications by several independent groups [4–11]. However, our group has been the leader in the translation of SHI to* in vivo* applications going back more than a decade [3]. In this paper, we review our experiences with* in vivo* SHI including a variety of transducer options and clinical applications.

## 2. Subharmonic Imaging on 2D Linear Arrays

*In vivo* SHI was first performed by implementing subharmonic frequency filters on a Logiq 9 scanner with a 7L probe (GE Healthcare, Milwaukee, WI). By transmitting at 4.4 MHz and receiving at 2.2 MHz, subharmonic time intensity curves were generated from canine renal vasculature [12]. These curves were then used to calculate tissue perfusion, using a nonradioactive isotope microbead assay as a reference standard [12]. SHI tissue perfusion estimates were found to correlate well in high perfusion states in the anterior of the kidneys (*r* = 0.73; *P* = 0.0001) [12]. This SHI setup was then used for a clinical pilot study for characterizing breast lesions in women [13]. In 14 women (16 total lesions) receiving intravenous injections of either Definity (Lantheus Medical Imaging, North Billerica, MA) or Optison (GE Healthcare, Princeton, NJ), SHI was found to significantly improve enhancement of the mass relative to contrast-enhanced Power Doppler (100% versus 44% of lesions with good or excellent enhancement; *P* = 0.004). However, no statistically significant improvements in the area under receiver operating characteristics curve (Az) were observed between SHI (Az = 0.78) and any ultrasound mode for mass characterization (*P* > 0.2); [13], until postprocessing motion compensation and maximum intensity projections were applied (SHI Az = 0.90; *P* = 0.03 relative to mammography; [14]). Quantitative analysis has also since been applied to this dataset, showing that parametric analysis of mass perfusion [15] and vascular skeletonization [16] may also be useful parameters for characterizing breast lesions when using SHI.

SHI has also been implemented on other linear arrays by our group for research purposes. Recently, we implemented SHI on a Sonix RP scanner with a L9-4 probe (Analogic Ultrasound, Richmond, BC, Canada). SHI was performed using a variety of settings including transmitting 6.7 and 10 MHz. This setup was then used to estimate tumor interstitial pressure in swine [17] and angiogenic marker expression in a murine breast cancer model [18] with Definity. Rat tumors were also scanned with a Vevo 2100 (Visualsonics, Toronto, Ontario, Canada) using a 24 MHz probe during injection of Definity [19]. Similar to work by Needles et al. [6], subharmonic images were constructed by the postprocessing acquired radiofrequency data on the Vevo 2100 using filters at the subharmonic [19]. Tumor fractional vascularity was then calculated using both high frequency (*f*_0_ = 24 MHz) and low frequency (*f*_0_ = 8 MHz) SHI and compared to angiogenic marker expression on pathology, with the strongest correlation observed between high frequency SHI and vascular endothelial growth factor expression [19]. These results demonstrate the feasibility of SHI on a variety of ultrasound scanners using a variety of frequency pairings.

## 3. Subharmonic Imaging on a Curvilinear Array

Pulse inversion subharmonic imaging has been implemented on a curvilinear probe on a Logiq 9 ultrasound scanner (GE Healthcare). This setup provides 4 cycle pulses transmitted on a 4C probe transmitting at 2.5 MHz and receiving at 1.25 MHz [20]. In addition, the experimental software transmits interleaved B-mode pulses to provide dual B-mode/SHI imaging [20]. This setup then allows the contrast-specific imaging of SHI with an ability to locate anatomical landmarks on B-mode ultrasound.

**Figure 1:**
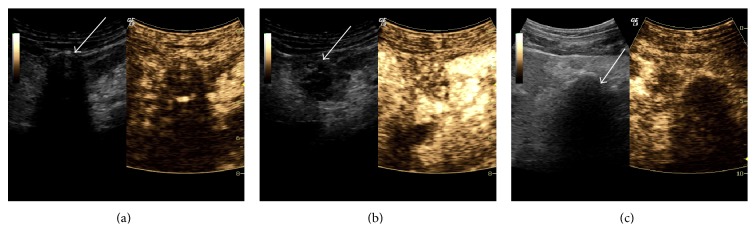
SHI exam of a 3.6 × 2.4 cm exophytic renal mass (arrow) at baseline (a), peak enhancement (b), and at peak enhancement 4 months after percutaneous cryoablation (c).

**Figure 2:**
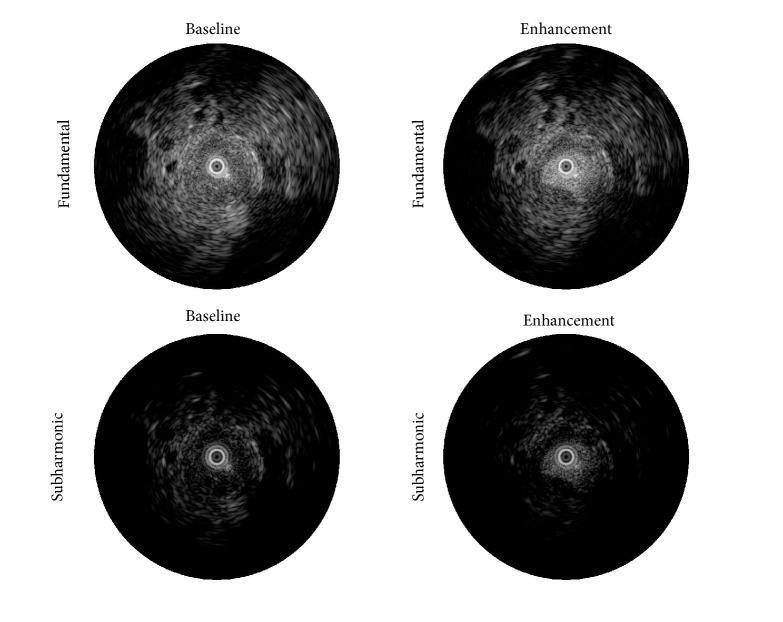
Intravascular ultrasound images of plaque in a rabbit model at baseline (left images) and during peak enhancement (right images) in both the original fundamental mode (top images) and SHI frequency filtered images (bottom images).

Our group has demonstrated the ability to perform SHI within the hepatic vasculature of both canines and humans using this curvilinear probe with the ultrasound contrast agent Sonazoid (GE Healthcare, Oslo, Norway) [20, 21]. More recently, our group has used this setup to image renal masses as a means for evaluating percutaneous cryoablation using the ultrasound contrast agent Optison (GE Healthcare, Princeton, NJ) [22]. An example from these studies is shown in Figure 1, depicting a 3.6 × 2.4 cm renal exophytic mass with calcifications on the right kidney of a patient prior to contrast injection (a), at approximate peak enhancement before cryoablation (b), and at approximate peak enhancement 4 months after cryoablation (c). Clear enhancement is visible within the mass and renal cortex before treatment (Figure 2 middle), while enhancement is only visible within the renal cortex after cryoablation (Figure 2 right) indicating effective ablation of the mass. Our group is currently investigating the ability to perform similar imaging studies in the pancreas as a means for characterizing pancreatic masses. Such results from the liver, kidneys, and pancreases demonstrate the ability of SHI to be performed at depths greater than 8 cm for abdominal applications.

## 4. Subharmonic Imaging Using Intravascular Ultrasound

Intravascular ultrasound is capable of providing real-time cross-sectional visualization of blood vessels at high resolution (100–150 *μ*m) [23], making it the preferred imaging modality for studying atherosclerosis. Characterizing vascular tissue and plaque composition is essential for determining the type of interventional procedure and subsequent pharmaceutical administration. However, the similar echogenicity of plaque and surrounding vascular tissue makes it hard to accurately differentiate between normal and atherosclerotic tissue. Our group investigated the ability to improve this delineation by isolating the subharmonic frequency response from Definity using a commercially available intravascular ultrasound system, Galaxy (Boston Scientific/Scimed, Marlborough, MA) [24, 25].

Contrast-enhanced intravascular ultrasound was performed on Watanabe Heritable Hyperlipidemic (WHHL) rabbits with atherosclerosis induced by a combination of high cholesterol diet and balloon deendothelization. Imaging was performed at a transmit frequency of 40 MHz (transmitting 2 cycle pulses) at a peak negative pressure of 5.6 MPa. The radiofrequency data was transferred to a desktop computer and filtered offline. Preliminary filters designed to isolate the subharmonic component (20 MHz) from the radiofrequency data were evaluated based on CTR and visual examination for image noise, plaque visualization, and vessel lumen visualization. Based on the outcomes, a subharmonic adaptive filter was developed along with a stopband filter (to suppress tissue signal around 40 MHz). Quantitatively, SHI had significantly higher vessel-plaque CTR than the fundamental (2.01 ± 2.21 versus 1.76 ± 2.28, *P* < 0.01), therefore producing the best plaque delineation [25]. An example of intravascular SHI from this study is shown in Figure 2, comparing the original acquired fundamental frequency data (top) and SHI filtered intravascular images (bottom) at baseline (left) and during peak enhancement (right). SHI provides improved tissue suppression relative to the fundamental, which leads to an improved delineation of the plaque neovascularity during contrast enhancement. Parametric images were also created from this dataset by constructing time intensity curves on a pixel by pixel basis. Using this analysis, it was shown that the generation of maximum intensity projections, perfusion, and time-integrated intensity (representing the area under the time intensity curve) further improved vessel-plaque CTRs for SHI relative to the fundamental and nonparametric SHI datasets (*P* < 0.04; [24]).

## 5. 4D Subharmonic Imaging

While results using 2D SHI in cancer imaging have been promising, tumor vasculature is often tortuous and heterogeneous, due to the erratic formation of blood vessels during angiogenesis [26, 27]. Thus, 2D imaging may fail to fully visualize the complete vascular architecture of these masses, which may also be useful for characterization. This line of reasoning has led us to the development of 4D or real-time 3D SHI in order to better visualize the complete vascular structure within a volume. Recently, 4D SHI was implemented on a 4D10L probe in combination with experimental software on a Logiq 9 scanner (GE Healthcare). This setup enables 4D pulse inversion SHI (transmitting 4 cycle pulses at 5.8 MHz and receiving at 2.9 MHz) and 4D pulse inversion HI (transmitting 2 cycle pulses at 5 MHz and receiving at 10 MHz) [28].

**Figure 3:**
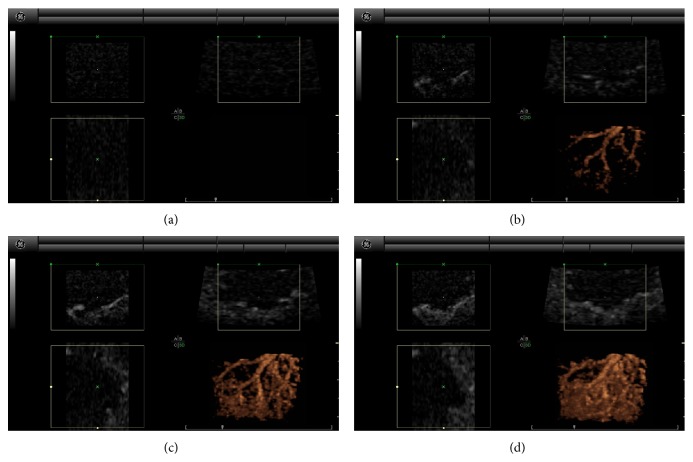
4D SHI example of canine renal vasculature during open abdomen scanning at baseline (a), during early contrast wash in (b), during later wash in (c), and during full enhancement (d).

Our group has demonstrated that sufficient volume acquisition rates can be generated with this equipment (1.8 to 2.2 volumes/second for 2.5 × 2.5 × 2.5 cm volumes) and that 4D SHI provides improved CTRs relative to 4D HI in both phantoms and canine kidneys (12.11 ± 0.52 versus 2.67 ± 0.77, *P* < 0.001* in vitro*, and 5.74 ± 1.92 versus 2.40 ± 0.48, *P* = 0.4* in vivo* [28].) An example of 4D SHI of canine renal vasculature during open abdomen scanning is shown in Figure 3. At baseline (Figure 3(a)), complete tissue suppression is evident. As contrast agent washes in to the renal vasculature (Figures 3(b) and 3(c)), a complete, connected representation of the larger renal vessels is demonstrated, before full enhancement of the entire kidney becomes apparent (Figure 3(d)). Our group has also investigated the ability of these modes to estimate tissue perfusion in a canine kidney model using a neutron labeled microsphere assay as a reference standard and found that 4D SHI provided a better overall estimate than either 4D HI or 2D SHI [29].

**Figure 4:**
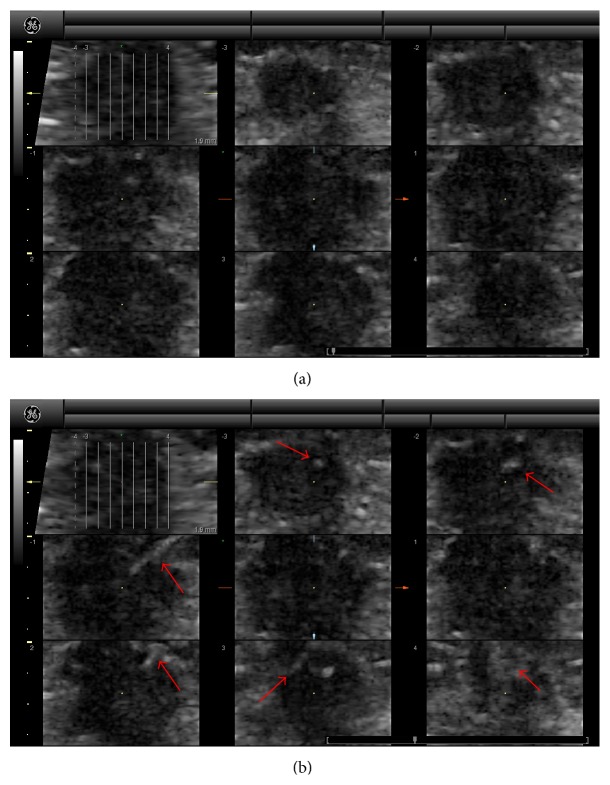
Example case of 4D SHI showing a 2.1 × 1.8 × 1.6 cm ductal invasive carcinoma* in situ* at baseline (a) and during contrast enhancement (b). Several feeding vessels can be observed (red arrows) during contrast enhancement.

Currently, our group is conducting a multicenter clinical trial with the University of California, San Diego, to investigate the use of 4D SHI and HI with this setup to characterize breast masses identified by mammography. Such a technique is hoped to reduce the large number of false-positive masses currently referred for biopsy after mammography. While this study is ongoing and the clinical utility of these techniques has yet to be determined, we have reported that 4D SHI provides improved tissue suppression in almost all cases to date and an improved ability to visualize vasculature within lesions [30]. An example case from this study is shown in Figure 4, showing a 2.1 × 1.8 × 1.6 cm ductal invasive carcinoma* in situ* (DCIS) at baseline (a) and during contrast enhancement (b). Identification of several feeding vessels (red arrows) can be seen in multiple imagine places during enhancement, demonstrating the importance of volumetric imaging.

## 6. Conclusions

The implementation of SHI has been shown to be feasible using a variety of ultrasound scanners and probes. The development of SHI on linear, curvilinear, intravascular, and 4D arrays has provided research avenues for a variety of clinical applications. These current research applications are expected to translate to clinical tools for improving patient care in the future.
